# Sildenafil ameliorates oxidative stress and DNA damage in the stenotic kidneys in mice with renovascular hypertension

**DOI:** 10.1186/1479-5876-12-35

**Published:** 2014-02-06

**Authors:** Ananda T Dias, Bianca P Rodrigues, Marcella L Porto, Agata L Gava, Camille M Balarini, Flavia P S Freitas, Zaira Palomino, Dulce E Casarini, Bianca P Campagnaro, Thiago M C Pereira, Silvana S Meyrelles, Elisardo C Vasquez

**Affiliations:** 1 Laboratory of Translational Physiology, Health Sciences Center, Federal University of Espirito Santo, Vitoria, ES, Brazil; 2 Department of Physiology and Pathology, Health Sciences Center, Federal University of Paraiba, João Pessoa, PB, Brazil; 3 Nephrology Division, Department of Medicine, Federal University of Sao Paulo, Sao Paulo, SP, Brazil; 4 Pharmaceutical Sciences Graduate Program, University of Vila Velha (UVV), Vila Velha, ES, Brazil; 5 Federal Institute of Education, Science and Technology (IFES), Vila Velha, ES, Brazil

**Keywords:** Sildenafil, DNA damage, Oxidative stress, Comet assay, Renovascular hypertension, Angiotensin, Stenotic kidney

## Abstract

**Background:**

Oxidative stress and DNA damage have been implicated in the pathogenesis of renovascular hypertension induced by renal artery stenosis in the two-kidney, one-clip (2K1C) Goldblatt model. Considering our previous report indicating that the chronic blockade of phosphodiesterase 5 with sildenafil (Viagra®) has marked beneficial effects on oxidative stress and DNA damage, we tested the hypothesis that sildenafil could also protect the stenotic kidneys of 2K1C hypertensive mice against oxidative stress and genotoxicity.

**Methods:**

The experiments were performed with C57BL6 mice subjected to renovascular hypertension by left renal artery clipping. Two weeks after clipping, the mice were treated with sildenafil (40 mg/kg/day for 2 weeks, 2K1C-sildenafil group) or the vehicle (2K1C). These mice were compared with control mice not subjected to renal artery clipping (Sham). After hemodynamic measurements, the stenotic kidneys were assessed using flow cytometry to evaluate cell viability and the comet assay to evaluate DNA damage. Measurements of intracellular superoxide anions and hydrogen peroxide levels as well as nitric oxide bioavailability were also obtained.

**Results:**

Sildenafil treatment significantly reduced mean arterial pressure (15%), heart rate (8%), intrarenal angiotensin II (50%) and renal atrophy (36%). In addition, it caused a remarkable decrease of reactive oxygen species production. On the other hand, sildenafil increased nitric oxide levels relative to those in the nontreated 2K1C mice. Sildenafil treatment also significantly reduced the high level of kidney DNA damage that is a characteristic of renovascular hypertensive mice.

**Conclusions:**

Our data reveal that sildenafil has a protective effect on the stenotic kidneys of 2K1C mice, suggesting a new use of phosphodiesterase 5 inhibitors for protection against the DNA damage observed in the hypoperfused kidneys of individuals with renovascular hypertension. Further translational research is necessary to delineate the mechanisms involved in the prevention of renal stenosis in the clinical setting.

## Background

Renal artery stenosis, the main cause of chronic renovascular disease, is associated with significant metabolic alterations in the kidney, such as increased renin synthesis and reduction of nitric oxide (NO) sensitivity and cGMP content [1,2], apoptosis and atrophy [3,4]. Recently, it has become evident that oxidative stress is one of the most important mechanisms involved in renal hypoperfusion. Oxidative stress may progressively impair renal function and contribute to irreversible renal damage [5,6]. Strategically, new pharmacological approaches have been developed that include vasodilators (e.g., atrial natriuretic peptide and low-dose dopamine), antioxidant enzyme mimetics and novel antioxidants [7,8]. However, these approaches have been unsuccessful in the clinical setting.

In the 1930s, a canine experimental model of unilateral renal artery stenosis (hypoperfusion), termed the 2K1C Goldblatt renovascular hypertension model, was developed. The model involved partially clipping the left kidney artery. Our laboratory has contributed to the understanding of the pathophysiology of renovascular hypertension using this model in rats since the 1990s [9-12] and more recently in mice [13-19]. It is an interesting disease model given the excessive production of oxidative stress, which can be explained by two major factors. First, endogenous activation of the renin secretion system results in augmented levels of plasma [3,18,19] and intrarenal [20,21] angiotensin II which is a potent stimulus for NADPH oxidase-induced reactive oxygen species (ROS) generation [6,22]. Second, based on experimental studies of ischemia/reperfusion injury, clip-induced hypoperfusion could result in microvascular damage characterized by oxidative stress-induced tissue injury, particularly when toxic oxidative species are involved [23,24].

In this context, alternative pharmacological strategies could be used to reduce oxidative stress and prevent molecular damage in the kidney. Recent data from our laboratory and others have indicated a potential application for sildenafil, a phosphodiesterase type 5 (PDE5) inhibitor, in many experimental models of diseases in addition to erectile dysfunction and pulmonary hypertension [25-28]. We have previously demonstrated that, in the atherosclerotic mice model, sildenafil reduces oxidative stress and increases NO bioavailability, which culminate in the protection against DNA damage [27,28]. However, the sildenafil’s efficacy in the treatment of chronic stenotic kidney in the renovascular hypertension model has not yet been investigated.

Therefore, the present study was designed to test the hypothesis that sildenafil decreases stenotic kidney damage in renovascular hypertensive mice by reducing oxidative stress and increasing NO bioavailability.

## Methods

### Animals

Experiments were performed in male wild-type (WT) mice (C57BL/6) that weighed 23 g on average (12-week-old). Mice were bred and maintained in the Laboratory of Translational Physiology animal facility (Vitoria, ES, Brazil) and were fed a standard chow diet and received water *ad libitum*. Animals were housed in individual plastic cages with automatic controlled temperature (22°C) and humidity (60%) and were exposed to a 12/12 h light–dark cycle. All of the experimental procedures were performed in accordance with the National Institutes of Health (NIH) guidelines, and the study protocols were approved by the Institutional Animal Care and Use Committee (CEUA-EMESCAM Protocol # 02/2013).

### Induction of 2K1C renovascular hypertension and treatment

The 2K1C angiotensin-dependent hypertension was induced as previously described [3,13,15,17] and recently reviewed by Campagnaro et al. [17]. Briefly, animals were anesthetized (91/9.1 mg/kg ketamine/xylazine, *i.p*.). The left renal artery was exposed through a retroperitoneal flank incision and carefully isolated from the renal vein, nerves, and connective tissues. Using an ophthalmic surgical microscope (Opto Eletronica SA, model SM 2002, Belo Horizonte, MG, Brazil), a U-shaped stainless steel clip with a 0.12 mm wide opening was placed around the renal artery near the abdominal aorta, which decreased renal perfusion [16,17]. Two weeks after surgery, animals were divided into two groups (8 to 10 animals per group): renovascular hypertensive mice treated with vehicle (2K1C) and hypertensive mice treated with 40 mg/kg/day of the PDE5-inhibitor sildenafil (Viagra®) for 2 weeks by oral gavage (2K1C-sildenafil). The effectiveness of this sildenafil dose was previously demonstrated in studies on endothelial dysfunction and DNA damage in our laboratory [27,28]. The dose was based on the fact that sildenafil displays reduced oral absorption by pre-systemic hepatic metabolism and high washout in mice [29]. Sham-operated mice were used as control animals (Sham).

### Hemodynamic measurements

Twenty-eight days after the 2K1C or sham operations, mice were anesthetized with a combination of ketamine/xylazine (91/9.1 mg/kg, i.p.), and a catheter (0.040 mm outer × 0.025 mm inner diameters, MicroRenathane, Braintree Science, Massachusetts, USA) was inserted into the right carotid artery to take mean arterial pressure (MAP) and heart rate (HR) recordings. The free end of the catheter was tunneled under the back skin to the level of the shoulder blades. Two days after the catheter placement, hemodynamic measurements were performed in conscious, freely moving mice in their cages, as already validated by others [30] and standardized in our laboratory [13-17] as a sufficient period for complete recovery from surgery. For the MAP and HR recordings, the arterial catheter was plugged into a disposable blood pressure transducer (Cobe Laboratories, Colorado, USA) connected to a pressure processor amplifier and data-acquisition system (MP100, Biopac Systems, California, USA). At the beginning of the experimental session, a period of approximately 30 min was allowed for stabilization of cardiovascular parameters before the measurement of basal MAP and HR values in conscious mice (Acknowledge software, Biopac Systems, California, USA).

### Biochemical analysis of renal function

Mice were placed in metabolic cages for a 24-hour adaptation period followed by another 24-hour period for biochemical analysis of urine. After, animals were euthanized, and their blood was collected for creatinine and urea measurements using commercial test kits (Bioclin®, BH, Brazil). Proteinuria was measured in urine samples by spectrophotometry (SP-220, Bioespectro, SP, Brazil) after endpoint reaction with a colorimetric kit (Sensiprot, Labtest Diagnostica S.A., Sao Paulo, Brazil).

### Measurements of angiotensin II in kidney tissue

Renal levels of angiotensin II were analyzed by HPCL. Briefly, angiotensin peptides were extracted from the left kidney sample homogenates and purified in Oasis C18 columns (Waters Corporation) previously activated with methanol (5 mL), tetrahydrofuran (5 mL), hexane (5 mL), methanol (5 mL) and water (10 mL). After activation, the samples were applied to the columns, washed with water and eluted in ethanol/acetic acid/water in the proportions of 90/4/6. The eluates were dried, redissolved in 500 μL of mobile phase A (5% acetonitrile in 0.1% phosphoric acid), filtered and injected into the HPLC system. Angiotensin peptides we analyzed by reversed-phase ODS Aquapore 300 (250 x 4.6 mm) HPLC column, 7 μm particle size (PerkinElmer’s Brownlee Columns), using the gradient 5–35% of mobile phase B (95% acetonitrile in 0.1% phosphoric acid) under a flow of 1.5 mL/min for 40 min. The angiotensin peptides were identified according to retention time (<6%) and peak height (<5%) of standard angiotensin peptides and normalized according to kidney weight.

### Cell samples and viability assay for flow cytometry

Kidney cell-enriched fractions from the kidneys of the mouse groups were prepared based on previous studies [31-33]. The left kidney was grossly triturated using surgical scissors and incubated with an extraction solution containing proteinase K (Sigma-Aldrich, St. Louis, MO, USA) and collagenase type II (Gibco Life Technologies, Sao Paulo, SP, Brazil) to dissociate the cells. The cell extract was filtered through a nylon screen (BD Falcon 70 μm) to remove the cell debris; the samples were washed twice in phosphate-buffered saline (PBS) and stored at −80°C until further analysis.

Cell viability was assessed by propidium iodide (PI) exclusion. A total of 10^6^ cells were incubated with 2 μL of PI for 5 min in the dark at room temperature. Then, cells were washed with PBS and analyzed with a FACSCanto II flow cytometer (Becton Dickinson). For viability quantification, samples were acquired in triplicate, and 10,000 events were used for each measurement. Cells were excited at 488 nm, and PI fluorescence was detected using a 585/42 bandpass filter. Data are expressed as the percentage of unstained/viable cells [34].

### Measurement of intracellular reactive oxygen species

The ROS analysis was performed by flow cytometry as previously described [18,19,35]. Dihydroethidium (DHE) and 2′,7′-dichlorofluorescein diacetate (DCF-DA) were used to detect intracellular •O_2_^–^ and H_2_O_2_, respectively. Given its ability to freely permeate cell membranes, DHE has extensively been used to monitor •O_2_^–^ production. Upon reaction with •O_2_^–^, DHE is rapidly oxidized to form ethidium, a red fluorescent product that intercalates DNA and amplifies the red fluorescence signal. DCF-DA is a cell permeant indicator of H_2_O_2_ production that is nonfluorescent until oxidation occurs within the cell, which converts DCF-DA into the fluorescent form, which remains trapped in the cell. DHE (160 mM) and DCF-DA (20 mM) were added to cell suspensions (10^6^ cells), which were then incubated at 37°C for 30 min in the dark, to determine the intracellular •O_2_^–^ and H_2_O_2_ concentrations, respectively [35]. Samples that were treated with 10 μM doxorubicin or 50 mM H_2_O_2_ for 5 min to create oxidative stress without cell toxicity, were used as the positive control. Cells incubated with ethanol were used as the negative control. The NO measurements were performed as previously described [36]. Briefly, the NO-sensitive fluorescent probe 4,5-diaminofluorescein-2 diacetate (DAF-2/DA: 2 μM) was added to the cell suspension (10^6^ cells), and the cells were incubated at 37°C for 180 min in the dark. Samples were treated with 10 μM sodium nitroprusside for the positive control. Cells were then washed, resuspended in PBS, and maintained on ice for immediate detection by flow cytometry (FACSCanto II, Becton Dickinson, San Juan, CA, USA). Data were analyzed using the FACSDiva software (Becton Dickinson), and overlay histograms were constructed using the FCS Express software. For fluorescence quantification samples were acquired in duplicate, and 10,000 events were used for each measurement. Cells were excited at 488 nm, and DHE, DCF and DAF fluorescence were detected using 585/42 (DHE) and 530/30 (DCF and DAF) bandpass filters. Data were expressed as the geometric mean fluorescence intensity (MFI).

### Measurement of oxidized DNA by the alkaline comet assay

The DNA damage was assessed using alkaline single cell gel electrophoresis (the alkaline comet assay). The technique was performed using established protocols from our laboratory [18,19,28,35] that were based on those of Singh et al. [37] with minor modifications. Given the thermo- and photo-sensitivity of the assay, the alkaline comet assay was performed under low brightness and controlled temperature.

The comet assay is a well-validated technique for DNA damage measurements in individual cells. In brief, histological slides were precoated with 1.5% normal melting point agarose. Subsequently, 20 μL of the cell suspension (containing 10^4^ cells) was embedded in 100 μL of 0.5% low melting point agarose and spread on agarose-precoated slides using coverslips. After agarose gelling, the coverslips were removed, and the slides were immersed in freshly prepared lysis solution (2.5 M NaCl, 100 mM EDTA, 10 mM Tris, 34 mM N-lauroylsarcosine sodium at pH 10.0-10.5 with freshly added 1% Triton X-100 and 10% DMSO) for 1 hour at 4°C. Then, the slides were placed in an electrophoresis chamber filled with freshly prepared alkaline buffer (300 mM NaOH, 1 mM EDTA, pH > 13) for 40 min at 4°C and electrophoresed at 300 mA and 20 V for 30 min. Next, the slides were neutralized with a 0.4 M Tris buffer (pH 7.5) for 5 min, washed with cold distilled water and dried at room temperature for 1 hour.

The migration of DNA fragments toward the anode creates a comet ‘tail’ that is visualized by staining with ethidium bromide (20 μg/mL, Sigma-Aldrich). Images were immediately obtained at 20× magnification using a fluorescence optical microscope (Nikon Eclipse Ti, Melville, NY, USA) equipped with excitation (510–550 nm) and barrier (590 nm) filters. The coded images were acquired using a CCD camera (Nikon) and analyzed with the CASP program (public domain). Among several parameters provided by the program CASP, we used the percentage of DNA in the tail and the tail moment for analysis of DNA damage. The images of 100 randomly selected cells from each sample obtained from each animal with two replicate slides were analyzed. During the image analysis, comets without clearly identifiable heads or comets with the majority of DNA localized to the tail after electrophoresis were excluded as a quality control parameter.

### Statistical analysis

Data are presented as either representative figures or the mean ± standard error of the mean (SEM). Flow cytometry data are expressed as the geometric mean (GeomMean) fluorescence intensity or the percentage of stained cells. The Kolmogorov-Smirnov test indicated that the variables displayed a normal (Gaussian) distribution. The statistical analysis was performed using one-way analysis of variance (ANOVA). When significant differences were demonstrated by ANOVA, the *post hoc* Bonferroni’s test was performed. The statistical analyses were performed using the Prism software (Prism 6.04, GraphPad Software, Inc., San Diego, CA, USA). The differences were considered significant when p < 0.05.

## Results

### Body and kidney weights, MAP and HR parameters and angiotensin II levels

**Figure 1:**
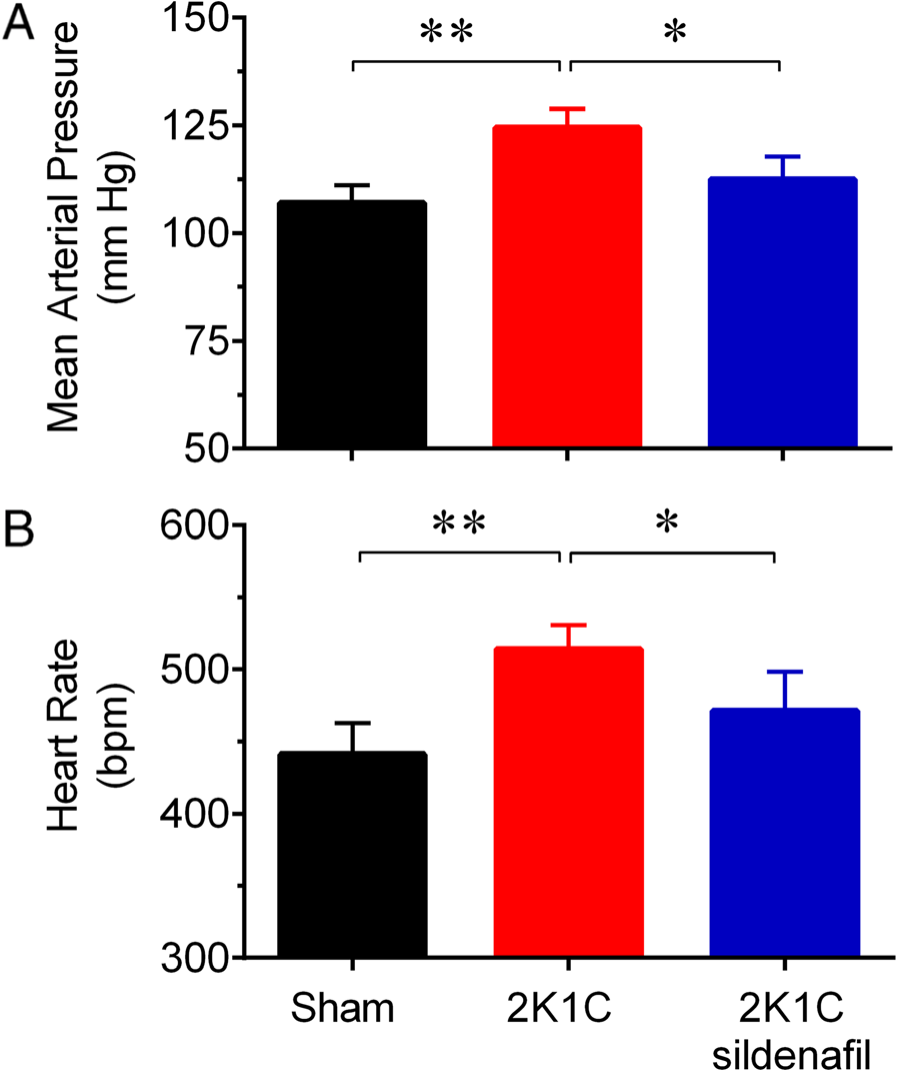
**Sildenafil reduces arterial hypertension in renovascular hypertensive mice.** Resting mean arterial pressure **(A)** and heart rate **(B)** values in conscious Sham (n = 8), 2K1C (n = 8) and 2K1C-sildenafil (n = 8) mice. Values are the means ± SEM. *p < 0.05 and **p < 0.01 (ANOVA).

**Figure 2:**
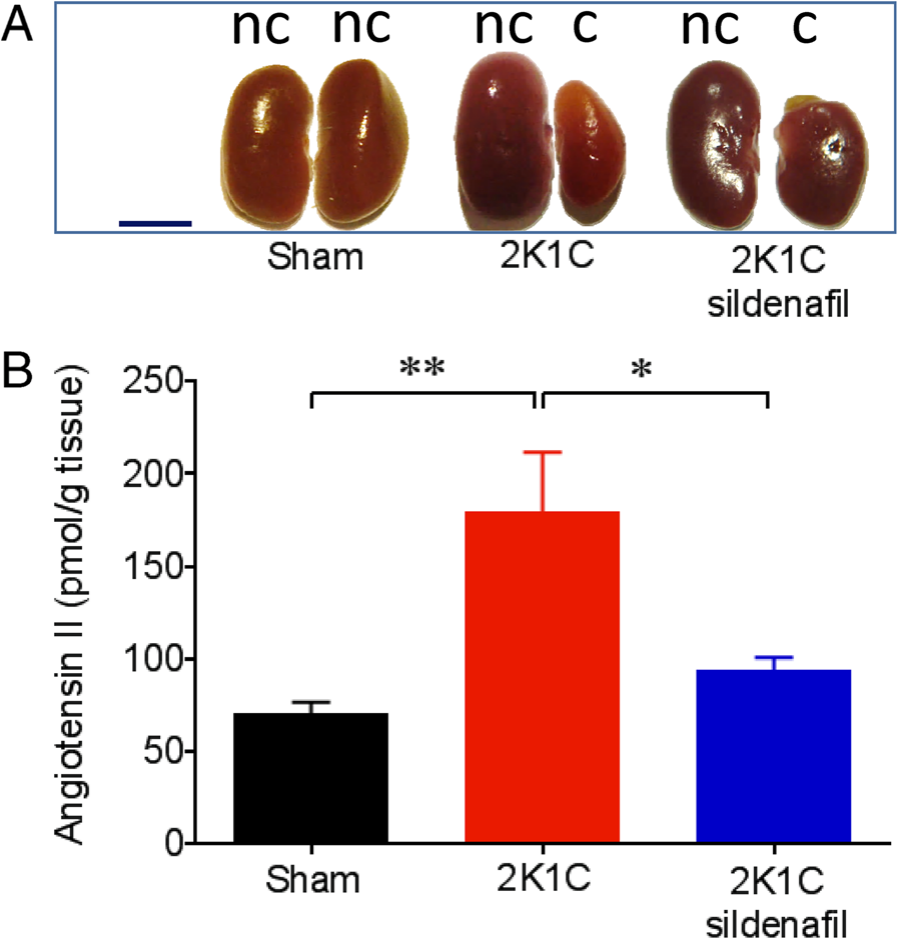
**Sildenafil treatment decreases angiotensin II levels in the stenotic kidney of renovascular hypertensive mice.** Panel **A**: typical photomacrograph showing the atrophy of the clipped (c) kidney and the hypertrophy of the nonclipped (nc) kidney caused by renal artery stenosis in the 2K1C mice compared with the Sham mice. Images were obtained 28 days after the surgery. Scale bar: 5 mm. Panel **B**: bar graph shows augmented levels of angiotensin II in the stenotic kidneys of the 2K1C group and the reduction of this peptide by sildenafil. Values are the means ± SEM, n = 8 to 10 per group. *p < 0.05 and **p < 0.01 (ANOVA).

Initial body weight was statistically similar among the groups. By the end of the experiments, only the 2K1C group displayed reduced body weight. Twenty-eight days after surgery, the left clipped kidney was atrophic, whereas the right nonclipped kidney displayed compensatory hypertrophy in the 2K1C mice. Interestingly, sildenafil not only reduced renal atrophy but also attenuated the compensatory hypertrophy (Table 1 and Figure 2A). Figure 1 shows the average values of resting MAP and HR measurements in conscious animals 28 days after renal artery clipping. As expected, the 2K1C mice showed higher MAP than the Sham mice (125 ± 2 vs. 107 ± 2 mmHg, p < 0.01), and the 2K1C mice treated with sildenafil showed MAP levels (112 ± 2 mmHg) statistically similar to those observed in the Sham mice (Figure 1A). The resting HR of the 2K1C mice was significantly higher than that in observed in Sham mice and sildenafil treatment abolished this tachycardia (Sham: 441 ± 10 bpm; 2K1C: 514 ± 7 bpm; 2K1C-sildenafil: 472 ± 15 bpm; p < 0.05) (Figure 1B). Figure 2 (panel B) shows average values of intrarenal angiotensin II in clipped kidneys in the 3 groups of animals. Angiotensin II levels in 2K1C mice were significantly augmented when compared with Sham mice (179 ± 32 vs. 70 ± 7 *p*mol/g tissue, p < 0.01). The 2K1C mice treated with sildenafil exhibited a reduction of 50% in these levels.

**Table 1:** Body and kidney weights of Sham, 2K1C and 2K1C-sildenafil treated mice 28 days after renal artery clipping

**Parameters**	**Sham (n = 8)**	**2K1C (n = 10)**	**2K1C sildenafil (n = 8)**
Body weight (g)	26 ± 0.5	24 ± 0.4*	26 ± 0.3^#^
Clipped kidney dry weight/body weight (mg/g)	1.63 ± 0.07	0.96 ± 0.17**	1.5 ± 0.04^#^
Nonclipped kidney dry weight/body weight (mg/g)	1.78 ± 0.04	2.02 ± 0.06*	1.77 ± 0.05^#^
Clipped kidney weight/nonclipped kidney weight ratio	0.91 ± 0.04	0.54 ± 0.09**	0.78 ± 0.06^#^
Kidney cell viability (%)	99 ± 0.13	94 ± 1.5**	98 ± 0.6^#^

Values are the means ± SEM. *p < 0.05 and **p < 0.01 compared with Sham group; ^#^*p <* 0.05 compared with 2K1C group.

### Renal function

The analysis of biochemical parameters showed that after 28 days of renovascular hypertension the kidney function was well preserved. We did not observe significant differences in plasma creatinine (0.44 ± 0.07 vs. 0.40 ± 0.05 mg/dL), urea (65 ± 5 vs. 61 ± 2 mg/dL), proteinuria (165 ± 23 vs. 145 ± 16 mg/dL/24 h) and the estimated glomerular filtration rate (8.2 ± 1.1 vs. 7.6 ± 1.8 μL/min) between groups. Sildenafil treatment did not modify those parameters in 2K1C mice.

### Cell viability assay

We evaluated the effect of clipping-induced stenosis on renal cell viability using flow cytometry of PI-labeled cells. As summarized in Table 1, the stenotic kidneys from the 2K1C group displayed reduced cell viability (Sham: 99 ± 0.13% vs. 2K1C: 94 ± 1.5%, p < 0.05), which was restored with sildenafil treatment (98 ± 0.6% viable cells).

### ROS production

**Figure 3:**
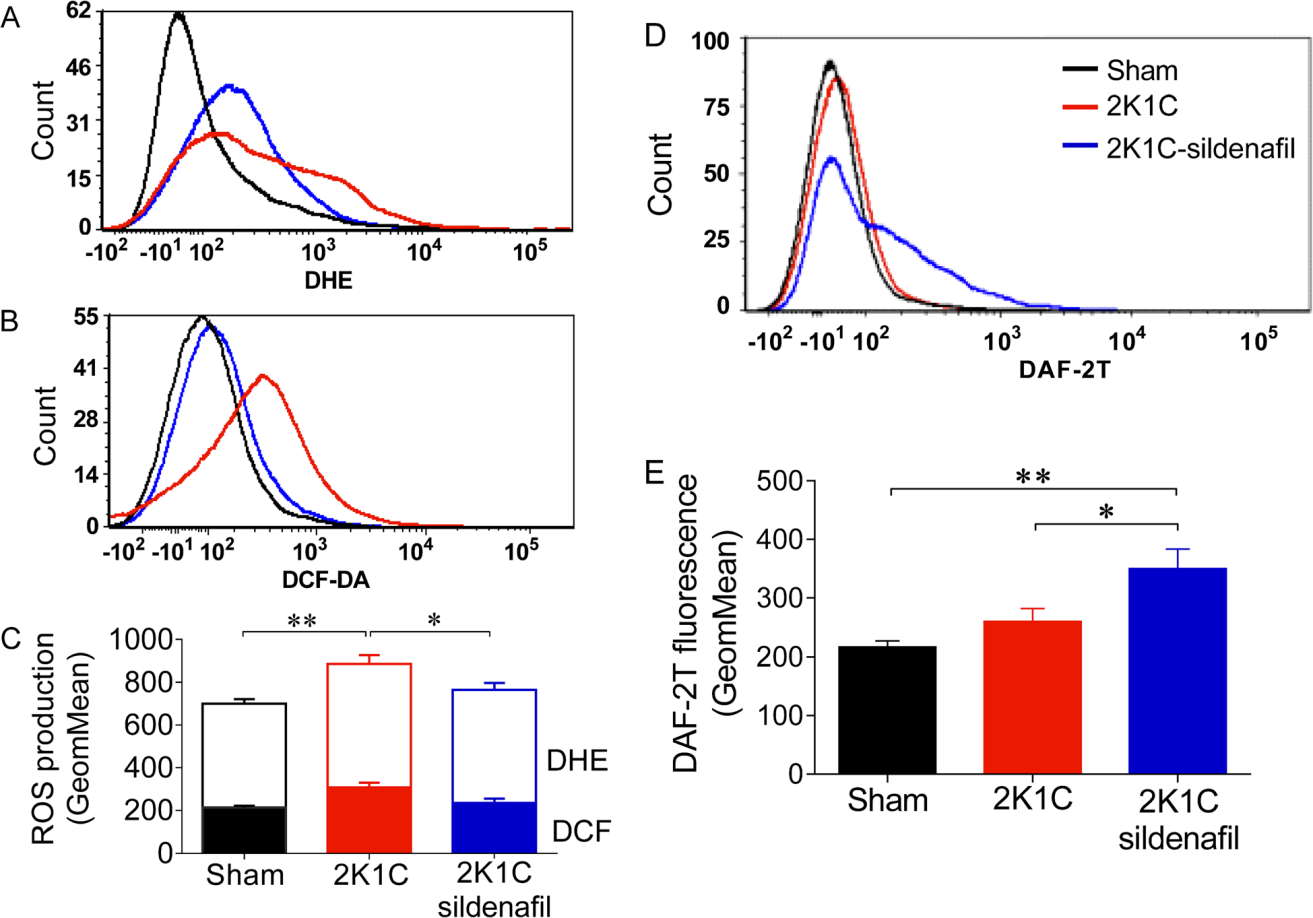
**Sildenafil ameliorates oxidative stress of the stenotic kidney cells in renovascular hypertensive mice.** Representative flow cytometry histograms show the fluorescence intensities of DHE (panel **A**), DCF-DA (panel **B**) and DAF-2/DA (panel **D**) stained cells from the Sham (n = 10), 2K1C (n = 10) and 2K1C-sildenafil (n = 10) mice. Bar graphs show the geometric mean intensity of DHE (panel **C**, white bars) and DCF-2/DA (panel **C**, color bars) and NO (panel **E**) production in the stenotic kidney. *p < 0.05 and **p < 0.01 (ANOVA).

ROS production was assessed using flow cytometry with DHE, DCF-DA and DAF-2/DA to quantify the production of •O_2_^–^, H_2_O_2_ and NO, respectively. Typical histograms from the flow cytometric analysis showed a rightward-shift in the log of DHE (Figure 3A) and DCF (Figure 3B) fluorescence in the 2K1C group compared with the Sham group, contrasting with the results of the 2K1C-sildenafil group. Differently, the typical histogram of NO production (Figure 3D), showed a rightward-shift in the log of DAF fluorescence in the 2K1C-sildenafil group compared with the Sham and 2K1C groups. As shown in Figure 3C, the 2K1C mice exhibited a remarkable increase (p < 0.05) in •O_2_^–^ (887 ± 41 a.u.) and H_2_O_2_ (308 ± 22 a.u.) levels compared with the Sham mice (700 ± 21 and 214 ± 8 a.u., respectively). Sildenafil treatment significantly decreased the •O_2_^–^ and H_2_O_2_ levels in the stenotic and hypoperfused kidneys (765 ± 32 and 235 ± 20 a.u., p < 0.05). Figure 3 (panel E) shows average values of DAF fluorescence in the stenotic kidney cells. Sham and 2K1C mice exhibited similar levels of NO (215 ± 9 vs. 260 ± 22 a.u.). The 2K1C mice treated with sildenafil displayed increased NO levels (1.7-fold, p < 0.01).

### Analysis of DNA damage by comet assay

**Figure 4:**
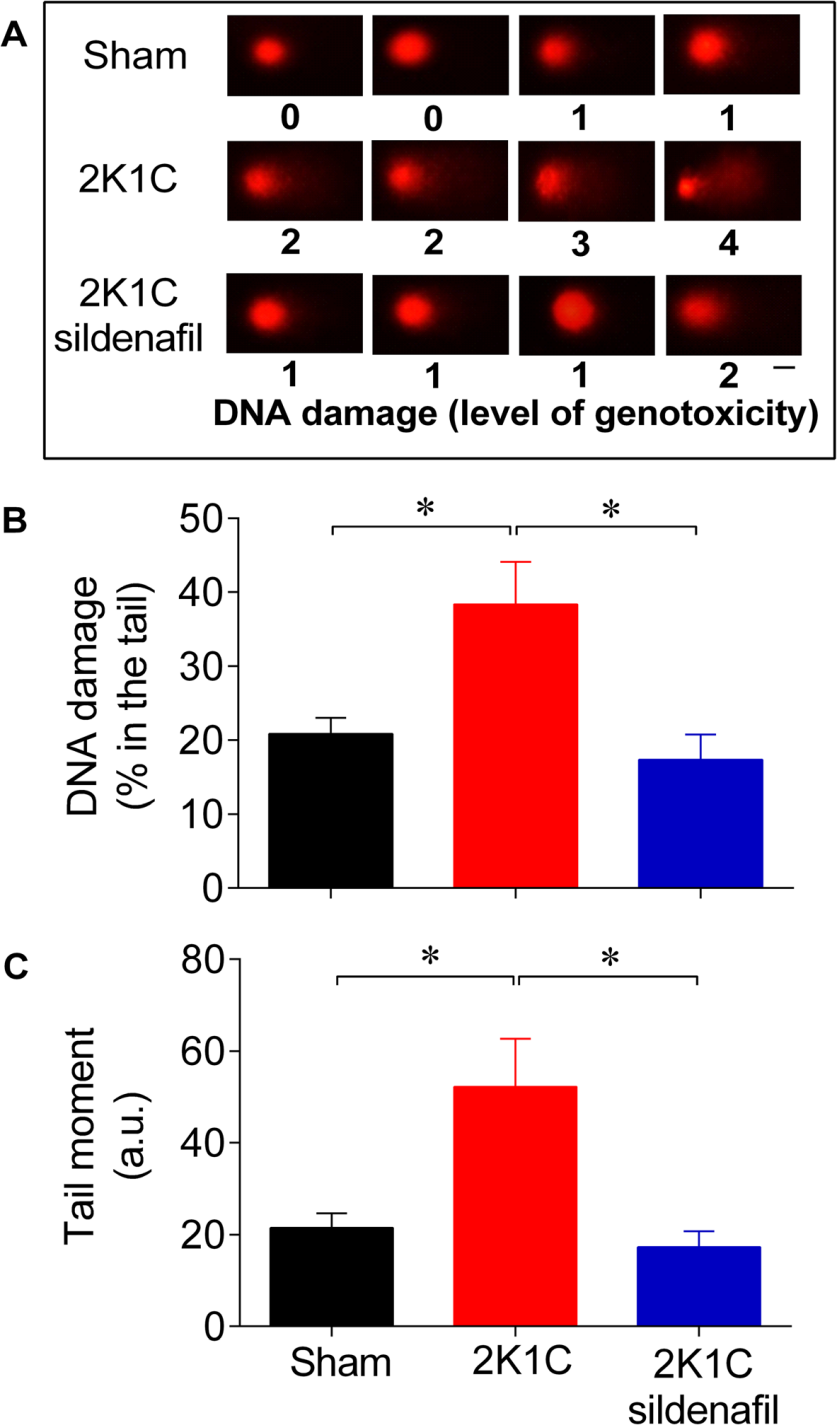
**Sildenafil restores DNA integrity of the stenotic kidney cells in renovascular hypertensive mice.** Panel **A**: Typical comets showing higher DNA fragmentation in the 2K1C hypertensive mice compared with the Sham mice and the beneficial effects of sildenafil in decreasing the DNA fragmentation. Levels of genotoxicity according to the comet tail size: 0 (intact comets), 1 (1% - 25% damage), 2 (26% - 45% damage), 3 (46% - 70% damage) and 4 (more than 70% damage). Scale bar: 10 μm. Bar graphs showing the mean percentage of DNA in tail (panel **B**) and the mean of the tail moment (panel **C**). Values are the means ± SEM, n = 10 per group. *p < 0.05 (ANOVA).

Figure 4A shows representative micrographs of the comets obtained from the stenotic kidney cells of each group. The images indicate no or minimal DNA fragmentation (0 and 1) in the Sham mice. In contrast, the representative images from the 2K1C hypertensive mice indicate high (3 and 4) levels of genotoxicity. Mice treated with sildenafil exhibited minimal DNA damage. Figure 4B shows the average percentage of fragments in the tail of the comet, which represents the degree of DNA damage in the 3 groups. The percentage of DNA in the comet tail represents the number of fragments that migrated during the electrophoresis. The 2K1C group exhibited a 2-fold increase in DNA fragmentation compared with the Sham group. The 2K1C mice treated with sildenafil exhibited minimal DNA damage, comparable to levels observed in the Sham mice. The comet tail moment, an index of both the migration of the genetic material and the relative amount of DNA in the tail, was analyzed [38]. As shown in Figure 4C, the cells from the stenotic kidney of 2K1C mice showed a significant increase of the levels of DNA damage compared with the Sham mice (52 ± 10 vs. 22 ± 3 a.u., p < 0.05). The DNA fragmentation in the cells isolated from the mice treated with sildenafil (17 ± 4 a.u.) was similar to the levels observed in the Sham group.

## Discussion

The present study is the first to report that the chronic inhibition of PDE5 with sildenafil decreases MAP, HR and renal damage in renovascular hypertensive mice. Beneficial effects of sildenafil include reduction of intrarenal angiotensin II and oxidative stress and enhanced NO bioavailability as well as a subsequent improvement of viability and a decrease of DNA damage in the stenotic kidney.

Current data indicate that •O_2_^–^ and H_2_O_2_ are significantly increased in the pathophysiology of ischemic renal diseases [39,40] and associated with DNA damage and apoptosis in the outer cells of 2K1C hypertensive animals [18,19]. Moreover, recent studies from our laboratory on oxidative stress-induced disease have demonstrated that sildenafil exhibits antioxidant effects, thereby preventing DNA damage in the liver and mononuclear cells [28]. These results strongly support the hypothesis that ROS play an important molecular role in renal and cardiovascular diseases (e.g. ischemic diseases, hypertension, atherosclerosis) [18,19,41]. Moreover, this self-perpetuating cycle leads to progressive target organ damage. Our rational for the present study was that sildenafil could also have beneficial effects on ROS in the clipping-induced stenotic kidney in the 2K1C angiotensin II-dependent hypertension.

Cumulative evidence suggests that PDE5 inhibition may be a suitable option for reducing arterial pressure in primary (essential) hypertension and may have additional benefits on endothelial dysfunction [42]. Our study extends this idea because it demonstrates sildenafil’s antihypertensive properties as well as its ability to restore HR and protect against renal damage under conditions of hypertension. These results reinforce recent data that sildenafil is an interesting alternative treatment for the primary cause of secondary hypertension [2]. Regarding the HR, previous studies from our laboratory showed that the chronic inhibition of NO synthesis increases the central sympathetic drive [43,44]. The present results emphasize this hypothesis since an improvement of sensitization of the NO/cGMP pathway by sildenafil has been reported by Stegbauer et al. [2], which could contribute to the normalization of HR. Considering the evidence that sildenafil crosses the blood–brain barrier and that PDE5 is present inside the brain [45], we cannot rule out the possibility that sildenafil could have a direct central influence on sympathetic/parasympathetic drive. In fact, recent results in rats have shown an increased sympathetic drive imposed on the cardiovascular system by sildenafil, which was not mediated by baroreflex [46]. However, additional studies are needed to evaluate this influence in mice.

It has been demonstrated that 2K1C hypertension is mainly initiated by the activation of the renin-angiotensin system rather than by impairment of renal function [3]. In agreement with others [3,47,48], we did not observe significant differences in serum parameters of renal function between 2K1C and Sham mice, presumably due to a compensatory increase in glomerular filtration rate, which appears to be sustained by the contralateral (unclipped) kidney [49]. The novelty of our study includes the finding that sildenafil provided a double beneficial effect in this pathophysiology. First, by reduction of intrarenal angiotensin II levels, which are normally augmented in stenotic kidney [20,21], and second, by preservation of renal function. These studies contrast with other experimental [5,50,51] and clinical studies of renovascular hypertension [52,53], which have demonstrated that inhibitors of angiotensin system (ACE inhibitors or AT1 antagonists) caused impairment of renal function due to the acute deterioration of the glomerular filtration rate [51,54].

The above data reinforce the findings of Welch et al. [5] who showed the antihypertensive effects of the antioxidant tempol, which ameliorated the oxidative stress, glomerular filtration rate and oxygen efficiency in the clipped kidneys in rats. Those authors have shown that, in contrast with the angiotensin II receptor blocker candesartan, tempol primarily exhibited antihypertensive properties without causing renal failure. Thus, we speculate that treatment targeting the correction of redox balance with sildenafil could also offer advantages over angiotensin II receptor antagonism as indicated by our finding that sildenafil decreases atrophy and increases cell viability in stenotic kidney possible by the mechanisms described below.

Given the reduced blood flow to the clipped kidney, oxygen deprivation and oxidative stress are unavoidable [55,56]. In this context, ROS overproduction reduces NO bioavailability in the renal vasculature via a scavenging effect and/or NOS uncoupling, leading to the increased production of •O_2_^–^ and H_2_O_2_[4,57-59]. The present study is the first to measure NO bioavailability in renal tissue from 2K1C using DAF-2/DA, which is a widely specific method for determination of NO levels in isolated cells from tissues. In contrast with plasma [60], NO levels in the stenotic kidney of 2K1C mice were not decreased, probably due to compensatory actions elicited by angiotensin II and hypoperfusion. This hypothesis is corroborated by findings from us and from others demonstrating an upregulation of nNOS in clipped kidneys in the 2K1C model [1] and an increase of renal eNOS activity in the angiotensin II infusion model [61]. In contrast with studies in 2K1C rats, in which sildenafil did not modify the low plasma NO levels [60], here we are demonstrating that in stenotic kidney from 2K1C mice sildenafil increases 1.7-fold the levels of NO and reduces the overproduction of •O_2_^–^ and H_2_O_2_.

Sildenafil, a PDE5 inhibitor, is a promising therapeutic alternative given that it prevents the breakdown of NO-driven cGMP and upregulates iNOS and eNOS [25], although it exerts protective effects in null mice for NOS isoforms [65]. Additionally, sildenafil increases the enzymatic activities of superoxide dismutase, catalase and glutathione peroxidase [28,62] as well as potentially restores NOS functionality [63], thereby acting as a potent vasodilator [27,64]. Our data show by the first time that in the 2K1C angiotensin II-dependent hypertension, the protective actions of sildenafil are not solely mediated through a reduction in the known molecular mechanisms of oxidative stress, but also through other pathways, including the reduction of intrarenal angiotensin II levels and, thus, contributing to attenuation of NADPH oxidase signaling [17,41]. Moreover, the increase in cGMP inhibits NADPH oxidase expression and activity, thereby reducing •O_2_^–^ and H_2_O_2_ production and consequently enhancing NO bioavailability [27,64]. This notion has also been corroborated by the present results demonstrating the effectiveness of sildenafil in increasing DAF expression (directly) and decreasing DHE/DCF and arterial pressure (both indirectly). Therefore, the several antioxidative properties of sildenafil may constitute additional mechanisms for the augmentation of the NO-mediated pathway in renovascular injuries.

Another novel finding of our study is the observation that sildenafil treatment reduces DNA damage in the stenotic kidney of renovascular hypertension mice, possibly by the same antioxidative mechanisms described above. Using the comet assay, it was possible to assess the effectiveness of sildenafil on kidney cell genotoxicity even for a short time interval (2 weeks). This finding reinforces the idea that sildenafil prevents tissue damage induced by oxidative stress as previously reported by our lab [28] and others [66,67], thereby opening the possibility of translational studies investigating the protection of DNA in hypoperfused organs in the clinic. For example, many clinicians continue to identify patients with a progressive loss of kidney function despite restoring blood supply to the kidney using suitable therapeutic interventions that immediately restore oxygen to tissues (administering clot dissolving drugs or using balloon catheters) [8,51,55,68]. Although this treatment is primarily beneficial, the reoxygenation of ischemic tissues has been shown to cause tissue damage or reperfusion injury in a phenomenon known as the “oxygen paradox”. In this phenomenon, ROS derived from reperfusion are responsible for post-ischemic tissue injury [8,69], and these molecules can damage cellular components, such as DNA, proteins, and lipids [35,39,70], compromising kidney integrity. Therefore, sildenafil may serve as an alternative therapy for renovascular disease, expanding the possibilities of unconventional use of PDE inhibitors therapy in patients with diseases of the urogenital tract involving the NO/cGMP cascade [71].

Recent studies suggest that renal ischemia rapidly mobilizes endothelial progenitor cells (EPCs), which provide renoprotection in acute kidney injury [51,72-75]. However, others have shown that the pro-oxidant *milieu* induced in the 2K1C model is accompanied by apoptosis, primarily of interstitial CD34^+^/KDR^+^ progenitor cells. These cells are presumably recruited to participate in kidney repair, thereby impairing renal self-regeneration [74]. In addition, Aleksinskaya et al. [76] proposed that hypertension impairs NO signaling in the bone marrow, causing inadequate mobilization of stem/progenitor cells. In this context, sildenafil seems to have a positive effect; a recent report shows that a sildenafil dose similar to that used in our study increases the number of bone marrow-derived EPCs in situations where oxidative stress is increased [77]. These EPCs may be involved in the reduction of ROS and apoptosis through cell therapy as recently observed by our group [57,78]. In the present study, we cannot reject the participation of EPCs in improving cell viability and reducing DNA damage. Therefore, the NO/cGMP pathway could constitute an attractive approach to rescue EPC function, offering new insights into anti-ischemic therapies.

Although our data have shown that sildenafil reduced angiotensin II, ROS and DNA damage in the clipped kidneys in 2K1C mice, a relative limitation of our study is that we analyzed these parameters in the stenotic kidney without differentiating possible differences between medulla and cortex.

## Conclusions

These results emphasize the role of increased oxidative stress in the pathogenesis of renal injury in renovascular hypertension. Moreover, the study highlights the beneficial effect of sildenafil in preserving stenotic kidneys. Further investigations are needed to establish the feasibility and efficacy of sildenafil in clinical settings of renal hypoperfusion.

## Abbreviations

•O2^–^: Superoxide anion; 2K1C: Two-kidney, one-clip; BP: Blood pressure; CASP: Comet assay software project; DAF-2/DA: 4,5-diaminofluorescein-2/diacetate; DCF-DA: 2′,7′-dichlorofluorescein diacetate; DHE: Dihydroethidium; DMSO: Dimethyl sulfoxide; EPC: Endothelial progenitor cells; H2O2: Hydrogen peroxide; HR: Heart rate; MAP: Mean aterial pressure; NADPH: Nicotinamide adenine dinucleotide phosphate; NO: Nitric oxide; PDE5: Phosphodiesterase type 5; PI: Propidium iodide; ROS: Reactive oxygen species.

## Competing interests

The authors declare they have no competing interests.

## Authors’ contributions

ALG, ATD, MLP and BPR carried out experimental analysis and acquisition of data, analysis and interpretation of the data and drafted the manuscript. MLP, CMB and BPC participated in the study’s design, supervision in the critical revision of the manuscript and carried out the experimental analysis. FPSF, ZP and DEC carried out the protocol of analysis of angiotensin II at the Federal University of Sao Paulo. TMCP participated in the supervision and in the critical revision of the manuscript. SSM and ECV contributed to the conception, design and supervision of the study and interpretation of data. All authors read and approved the final version of the manuscript.

## Authors’ information

The corresponding author ECV is a full professor at the Dept. of Physiological Sciences (UFES); got his PhD at the School of Medicine of the University of Sao Paulo (Brazil); was a visiting associate professor at the University of Iowa (USA, 1989–1991 and 1997–2000); has published more than 100 papers (including 14 in Hypertension), 10 chapters and/or books, and 10 reviews in the field of hypertension, atherosclerosis and polymorphism; has worked in collaboration with Virend Somers (Mayo Clinics Foundation), Alan Kim Johnson and Mark Chapleau (University of Iowa), Bernard Fleury (Hospital Saint-Antoine, Paris, France) and Eduardo M Krieger (Incor, Brazil); acted as secretary of the Brazilian Society of Hypertension for 4 years; was editor of physiology section of the Braz J Med Biol Res for 5 years; his H Index is 15. SSM is an associate professor at the Federal University of Espirito Santo (Brazil); got her PhD at the University of Iowa (USA) under the supervision of Mark W Chapleau and collaborating with Francois Abboud and Donald Haisted; she was the first to use transgenic mice in the research at her university and has published many full papers and reviews in high impact journals, including those from BioMed Central; her research is focused on hypertension and atherosclerosis using animal models. ALG, BPC, CMB and TMCP are young investigators who got their PhD at our laboratory and now are professor of physiology and medicine in private and public schools of medicine. ATD, BPR and MLP are PhD students under the supervision of SSM and ECV.
